# Global-scale random bottom pressure fluctuations from oceanic intrinsic variability

**DOI:** 10.1126/sciadv.adg0278

**Published:** 2023-07-21

**Authors:** Mengnan Zhao, Rui M. Ponte, Thierry Penduff

**Affiliations:** ^1^ Atmospheric and Environmental Research Inc., Lexington, MA, USA.; ^2^ Université Grenoble Alpes, CNRS, INRAE, IRD, Grenoble INP, Institut des Géosciences de l’Environnement (IGE), Grenoble, France.

## Abstract

Intrinsic processes such as mesoscale turbulence have recently been proved as important as atmospheric variability in causing variations in ocean bottom pressure (*p*_b_). Intrinsic processes are also known to generate random variability on scales larger than the mesoscale through inverse energy cascades or large-scale baroclinic instability. Here, model analyses reveal a truly global-scale, intrinsic *p*_b_ mode of variability at monthly time scales that relies on a different mechanism. The intrinsic mode has largest amplitudes around Drake Passage and opposite polarity between the Southern Ocean and Atlantic/Arctic oceans. Its signature is consistent with localized eddy-driven *p*_b_ anomalies of opposite sign near Drake Passage that then adjust freely in the rest of the ocean via barotropic wave processes. This intrinsic mode seems consistent with observed *p*_b_ variability.

## Teaser

Small-scale intrinsic variability can lead to global-scale signals in ocean bottom pressure via fast wave adjustment processes.

## INTRODUCTION

Ocean bottom pressure (*p*_b_) provides an important metric to evaluate the global ocean mass distribution, which can change due to freshwater fluxes from the land and atmosphere and redistribution by the ocean circulation and tides (*1*). Better understanding of *p*_b_ and its dynamics provides insights on sea level changes, ocean circulation patterns, heat and freshwater budgets, and more generally on climate (*2*–*4*).

Global-scale measurements of *p*_b_ variations are available since the launch of the Gravity Recovery and Climate Experiment (GRACE) and its follow-on (GRACE-FO) satellites (*5*–*8*). Effective interpretations of *p*_b_ fields derived from these missions are essential for their proper assimilation in ocean models (*9*, *10*) and to better understand dynamics associated with *p*_b_ in relation to other climate variables.

Variations in *p*_b_ occur over a wide range of spatiotemporal scales, reaching thousands of kilometers. Many past studies thus implicitly assume that large-scale *p*_b_ variations are mostly a response to atmospheric fluctuations (*11*–*13*). Variations in *p*_b_ not only can indeed be forced directly by the atmospheric variability (e.g., surface wind stress) but can also emerge spontaneously from oceanic intrinsic processes (*14*, *15*); one thus needs to distinguish between forced and intrinsic bottom pressure variations (respectively denoted as $p_{b}^{f}$ and $p_{b}^{i}$ hereafter).

Nonlinear mesoscale (or smaller scale) turbulence is the best-known example of these intrinsic processes, which generate random $p_{b}^{i}$ variations at spatial scales of order 100 km and time scales of weeks to months. However, recent modeling studies have shown that $p_{b}^{i}$ variations could be as important as those of $p_{b}^{f}$ at scales of order 1000 km (*13*, *16*). In the intra-annual frequency band, $p_{b}^{i}$ can actually be larger than $p_{b}^{f}$ in almost a quarter of the ocean area (*16*).

Two main processes have been proposed to explain the existence of intrinsic variability at large spatiotemporal scales. One process involves a spatiotemporal inverse energy cascade, in which the kinetic energy of mesoscale turbulence nonlinearly feeds larger spatial and longer time scales (*17*–*19*). Another process is large-scale baroclinic instability, where horizontal density gradients of the general circulation feed large-scale random intrinsic variability (*20*, *21*). The present study reveals a truly global-scale mode of $p_{b}^{i}$ variability that involves a different mechanism to attain its coherence across several ocean basins.

## RESULTS

### Global-scale $p_{b}^{i}$ pattern

Disentangling $p_{b}^{i}$ and $p_{b}^{f}$ variability is barely possible from observational data or from the output of single-model simulations. Here, we take advantage of the large ensemble of eddy-permitting global ocean/sea-ice simulations from the Oceanic Chaos-Impacts, Structure, Predictability (OCCIPUT) project (*22*, *23*). A total of 50 ensemble members were initialized from a 21-year common spin-up, and then, they were driven between 1960 and 2015 by the same atmospheric forcing derived from the ERA-Interim atmospheric reanalysis (*22*). As explained in (*22*) and detailed in (*24*, *25*), these stochastic perturbations slightly affect density gradients, hence geostrophic velocities, within each member during year 1960; they trigger the growth of the ensemble spread, whose subsequent saturation and evolution is solely controlled by the unperturbed ocean dynamics. This ensemble yields 50 distinct realizations of this 56-year oceanic evolution and, particularly, of daily $p_{b}^{i}$ variability. Over most of the oceans, an ensemble of 50 members is sufficient to distinguish forced from intrinsic variability (*23*, *26*).

The daily *p*_b_ signals are averaged to monthly fields and within 3^β^ × 3^β^ cells to smooth out mesoscale features. The 3^β^ × 3^β^ cells are chosen to be consistent with GRACE data resolution, which allows for more convenient comparison and interpretation. At a given location and month, the forced signal $p_{b}^{f}$ is estimated by the ensemble mean of *p*_b_, and the intrinsic signal $p_{b}^{i}$ is derived from each ensemble member by subtracting $p_{b}^{f}$ from *p*_b_, giving 50 realizations of the $p_{b}^{i}$ field.

**Fig. 1.**
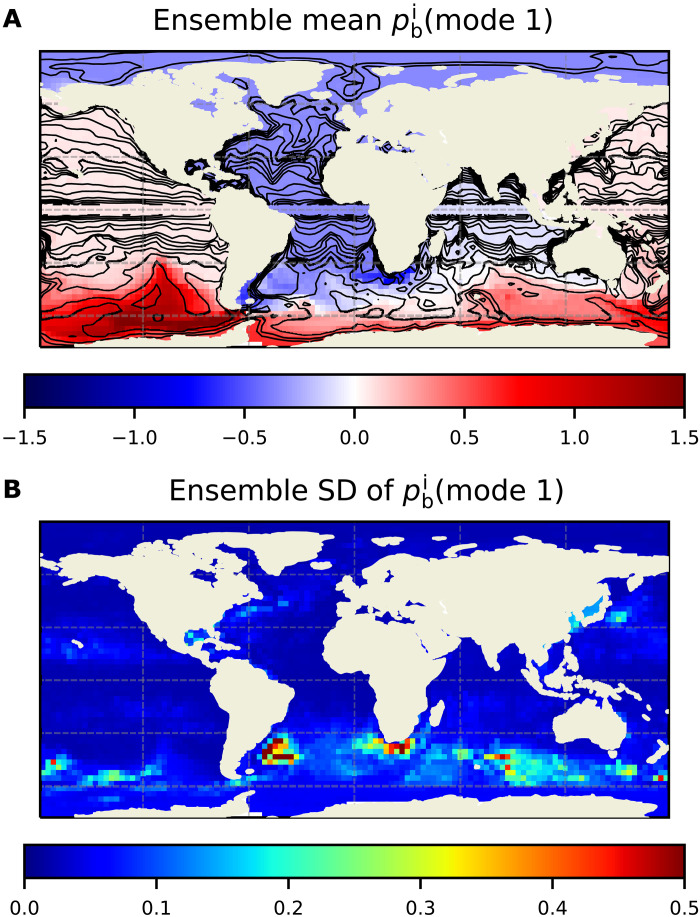
Global-scale EOF mode 1 of $p_{b}^{i}$ variability. Ensemble mean (**A**) and ensemble SD (**B**) of mode 1 $p_{b}^{i}$ variability across the 50 ensemble members. Units are in centimeter, where we have converted $p_{b}^{i}$ values to equivalent water thickness by dividing them by a reference sea water density and the acceleration of gravity, with 1 cm approximately equivalent to 1 hPa. Contours in (A) are lines of constant *H*/*f* [m·s].

To identify any potential large-scale spatial pattern, we apply empirical orthogonal function (EOF) decomposition to each of the 50 $p_{b}^{i}$ fields. We find a common leading EOF spatial pattern (mode 1 hereafter), with small SD over all ensemble members (Fig. 1). (Note that the leading EOF spatial patterns for different members could show opposite signs. Signs of the EOF patterns were made consistent before computing the ensemble average shown in Fig. 1A.) The common mode 1 explains, on average, 20.3% (±1.6%) of $p_{b}^{i}$ variance over all ensemble members. The $p_{b}^{i}$ variations in mode 1 exhibit a global-scale signature with out-of-phase behavior between the Southern Ocean and the Atlantic/Arctic oceans, where the EOF loading is largest (Fig. 1A), and are most prominent on monthly time scales (fig. S1).

**Fig. 2.**
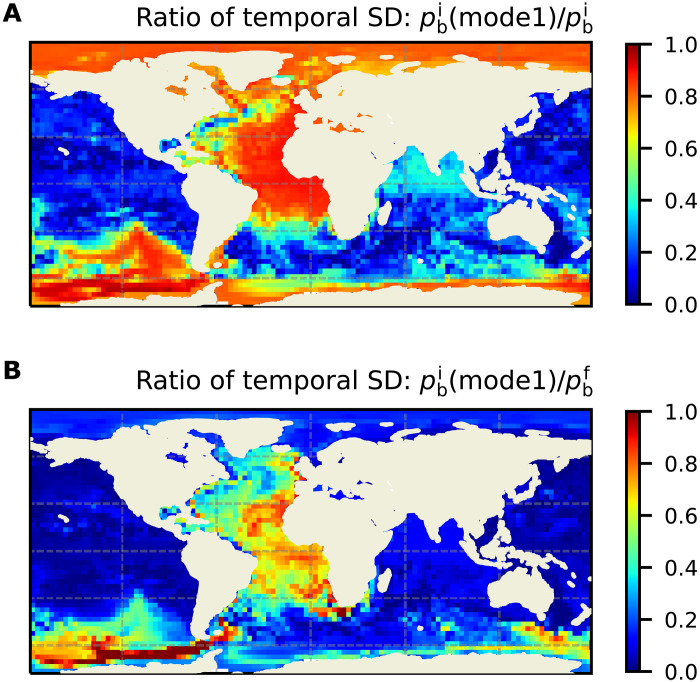
Importance of mode 1 $p_{b}^{i}$ variability. Ratio of the temporal SD of the mode 1 $p_{b}^{i}$ to that of total $p_{b}^{i}$ (**A**) and of $p_{b}^{f}$ (**B**) using one arbitrary ensemble member.

The amplitude of $p_{b}^{i}$ mode 1 reaches values of more than 1 cm in the Southern Ocean, with largest values around the Drake Passage region and some evidence for trapping of energy to the Antarctic coast, and very uniform values of several millimeters in the Atlantic and Arctic oceans (Fig. 1A). To quantify the importance of this global-scale $p_{b}^{i}$ pattern relative to other variability, we examine the ratio of the temporal SD of $p_{b}^{i}$ from mode 1 to those of total $p_{b}^{i}$ and $p_{b}^{f}$ for an arbitrary ensemble member (Fig. 2). (Results are not sensitive to the choice of ensemble member.) In the basins with largest amplitudes, mode 1 can amount to more than 80% of total $p_{b}^{i}$ variability (Fig. 2A) and more than 50% of total $p_{b}^{f}$ variability (Fig. 2B), indicating the importance of the global scale $p_{b}^{i}$ pattern highlighted in Fig. 1A for explaining *p*_b_ variability in vast areas of the ocean.

### Origin of the global-scale $p_{b}^{i}$ pattern

The emergence of a coherent pattern of $p_{b}^{i}$ variability on the planetary scale from nonlinear mesoscale processes would be unexpected. Although inverse cascade processes do not set a formal limit on the largest scales to which small-scale energy can be transferred (*18*, *19*), it is highly unlikely that these processes would give rise to some of the mode 1 features seen in Fig. 1 (e.g., homogeneous amplitudes within most basins but quite different across basins and with a clear global bipolar structure). Similar issues arise if one considers an explanation in terms of large-scale baroclinic instabilities, which also typically give rise to variability at (multi-)decadal scales (*20*, *21*) compared to the mostly subannual variability associated with mode 1 (fig. S1). Instead, the spatial character of mode 1 suggests a much more plausible interpretation, in terms of local generation of relatively small-scale $p_{b}^{i}$ anomalies, which then lead to a “free” barotropic adjustment over the global ocean.

Figure 1 shows the strongest amplitudes of mode 1 occur in regions near the Drake Passage, including the Bellingshausen-Amundsen basin and the Patagonian shelf. (There is also a maximum in the Agulhas retroflection region, but as per Fig. 1B, the amplitude of mode 1 in that region is more uncertain across the ensemble.) Moreover, the structure across Drake Passage is bipolar. Our interpretation is that relatively short-scale nonlinear processes generate relatively large $p_{b}^{i}$ anomalies of opposite sign across Drake Passage and that these localized anomalies constitute the main driving for the global mode. The $p_{b}^{i}$ signals in the rest of the ocean are essentially freely evolving barotropic adjustments to the $p_{b}^{i}$ anomalies across Drake Passage.

Anomalies to the west of Drake Passage adjust westward along *H*/*f* contours (*H* is water depth, and *f* is Coriolis parameter; Fig. 1A), which define pathways of propagation of oceanic barotropic Rossby waves (*27*, *28*) around Antarctica. In addition to Rossby-type barotropic adjustment, westward propagating Kelvin waves around this continent may also be involved. This adjustment is consistent with enhanced amplitude of mode 1 in the Bellingshausen-Amundsen basin and also around the Antarctic coast.

In contrast, anomalies on the Atlantic side adjust initially along South America and the equator through barotropic Kelvin wave propagation, followed by poleward propagation along the African and European coasts and into the Arctic. There is also adjustment from the eastern boundary through Rossby wave radiation along *H*/*f* contours (Fig. 1A). The end result is a quasi-equilibrium response with very weak $p_{b}^{i}$ gradients in the Atlantic/Arctic basins (Fig. 1A), given the fast adjustment time scales and the large spatial scales of the wave processses.

Barotropic waves involved in the global adjustment are indeed quite fast. Long Rossby wave and Kelvin wave propagation speeds scale as β*R*^2^ and $\sqrt{gH}$, respectively, where β ∼ 10^−11^ s ^−1^*m*^−1^ is the Rossby parameter and $R∼\sqrt{gH}/f∼2\;\cdot 10^{6}$ m is the barotropic Rossby deformation radius (*g* is acceleration of gravity) (*27*, *28*). With propagation speeds on the order of 200 m/s (Kelvin) and 40 m/s (Rossby), signals can cross ocean basins in a few days, i.e., much shorter than a month, consistent with our findings of the global-scale $p_{b}^{i}$ structure in Fig. 1.

In the Indian and Pacific oceans, mode 1 magnitudes are comparatively weak (Fig. 1A). We speculate that energy transmission from the Atlantic to the Indian Ocean around South Africa is not very efficient and that most of the energy propagating southward along the west coast of Africa ends up being scattered westward and staying in the Atlantic basin. Similarly, because westward propagating energy in the Southern Ocean is more confined to Antarctica, leakage of energy into the Pacific basin along the east coast of Australia is relatively weak. As a result, in both the Indian and Pacific oceans, mode 1 magnitudes can be substantially lower than those in the other basins.

### Global-scale $p_{b}^{i}$ pattern in GRACE observations

**Fig. 3.**
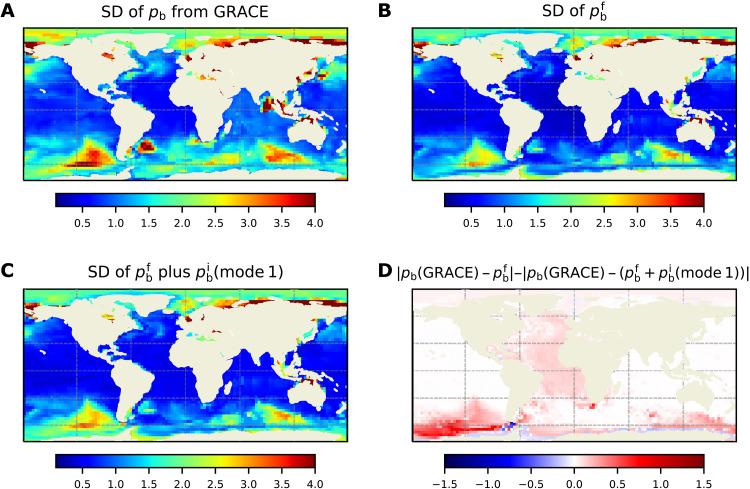
Global-scale $p_{b}^{i}$ in GRACE data. SD of *p*_b_ from GRACE (**A**), of $p_{b}^{f}$ from OCCIPUT (**B**), and of $p_{b}^{f}$ plus $p_{b}^{i}$ from mode 1 (**C**) using one arbitrary ensemble member. (**D**) Difference between the absolute value of (A) minus (B) and that of (A) minus (C). Units for all panels are in centimeter, as in Fig. 1.

The total *p*_b_ variability in OCCIPUT is consistent with that inferred from GRACE and GRACE-FO data (fig. S2). Given the global scales and amplitudes involved, the $p_{b}^{i}$ mode of variability derived from the OCCIPUT model analysis should be observable with GRACE and GRACE-FO measurements. Because there is no clear separation of scales between mode 1 and $p_{b}^{f}$, attempts to isolate this mode in the remotely observed *p*_b_ fields from those missions proved too challenging. Instead, to explore the presence of mode 1 in the GRACE observations, we compare the measured *p*_b_ variability (Fig. 3A) with two OCCIPUT-generated *p*_b_ fields: $p_{b}^{f}$ (Fig. 3B) and $p_{b}^{f}$ plus $p_{b}^{i}$ from mode 1 (Fig. 3C).

Over most of the oceans, we find that adding $p_{b}^{i}$ from mode 1 to $p_{b}^{f}$ brings the SDs simulated by OCCIPUT closer to those estimated from GRACE (Fig. 3D). Regions with largest improvements to modeled *p*_b_ variability when adding $p_{b}^{i}$ from mode 1 (e.g., the Bellingshausen-Amudsen basin) are coincident with the largest amplitudes in the mode 1 $p_{b}^{i}$ pattern (Fig. 1A), which are considerably larger than the noise level in GRACE data. Results in Fig. 3 indicate that the global-scale $p_{b}^{i}$ mode we identified from the model output is likely present in the observations.

## DISCUSSION

Our findings show the notable large-scale impacts on the *p*_b_ field of random intrinsic processes. The global-scale $p_{b}^{i}$ signature identified in this work is also reflected in the substantial $p_{b}^{i}$ variations found in relatively quiet eddy areas, such as the eastern Atlantic Ocean (*16*). Our analyses indicate that such global-scale $p_{b}^{i}$ variability can amount to at least half of $p_{b}^{f}$ variability in those regions and can thus be important for understanding *p_b_* variability in extensive ocean regions.

The existence of these global-scale pressure fluctuations from intrinsic processes in observations raises questions about being able to differentiate between $p_{b}^{i}$ and $p_{b}^{f}$. This is particularly important when comparing or assimilating GRACE-like observations with coarse-resolution models, in which *p*_b_ is mostly driven by atmospheric forcing, and is also relevant for trying to understand ocean variability and predictability more generally. Separation of $p_{b}^{i}$ and $p_{b}^{f}$ variability might be a difficult task, however, given possible mingling spatiotemporal scales, as is the case with the $p_{b}^{i}$ mode in Fig. 1A. We note that separating intrinsic from atmospherically driven signals discussed here is different from the topic of isolating anthropogenically forced signals from natural variability (*29*). The latter topic is potentially easier due to the distinct time scales in the two components.

Although the quasi-free global barotropic adjustment offers a plausible explanation for the basin-scale features of the $p_{b}^{i}$ mode highlighted here, the detailed nature of the “noise maker” in the Drake Passage region remains to be described. Instabilities of the Antarctic Circumpolar Current fronts could lead to related anomalous currents and mass transports. Details of the interactions of the mean flows with topography could be important. Moreover, location of instabilities and associated mass anomalies relative to the coasts and the structure of *H*/*f* contours, including regions of closed contours, could help define propagation pathways and the nature of the large-scale adjustment. These issues merit future dedicated model studies involving more variables than just *p*_b_ analyzed here and assessing sensitivities to model settings.

## MATERIALS AND METHODS

### GRACE and GRACE-FO *p*_b_ data

We use monthly *p*_b_ data returned from GRACE and GRACE-FO missions and processed by the Jet Propulsion Laboratory (RL06M.MSCNv02). The GRACE/GRACE-FO data are available from April 2002 to present, with horizontal resolution of 3°. For comparison with OCCIPUT output, the linear trend of *p*_b_ from GRACE/GRACE-FO on each grid point is removed.

### Isolating $p_{b}^{i}$ from $p_{b}^{f}$ using OCCIPUT output

Our analyses are based on the daily *p*_b_ output from 50 ensemble members from OCCIPUT (https://meom-group.github.io/projects/occiput/). The horizontal resolution is ∼1/4°, providing NEMO-based eddy-permitting ocean/ice hindcasts over 1960–2015. The ensemble members are driven by the same 6-hourly realistic atmospheric forcing from atmospheric reanalyses (Drakkar Forcing set DFS5.2). The linear trend of the *p*_b_ field is subtracted at each grid point within individual ensemble members to remove the potential contributions of model drift and geophysical tendencies and to focus on bottom pressure variability.

In this study, we analyze *p*_b_ over 2002–2015, the common period between OCCIPUT and GRACE. Daily, 1/4°-resolution *p*_b_ is first averaged to monthly and 3° × 3^β^ cells to obtain *p*_b_(*x*, *y*, *mon*, *mem*), where (*x*, *y*) denotes the grid cell, *mon* means the month, and *mem* represents the ensemble member. We can then calculate $p_{b}^{f}$ as the ensemble-mean pressure field $p_{b}^{f}(x,y,mon)=\sum_{mem=1}^{50}p_{b}(x,y,mon,mem)/50$. For individual ensemble members, $p_{b}^{i}$ is estimated as $p_{b}^{i}(x,y,mon,mem)=p_{b}(x,y,mon,mem)-p_{b}^{f}(x,y,mon)$.
