# Supplementary material for: Global-scale random bottom pressure fluctuations from oceanic intrinsic variability
Source: Sci Adv. 2023 Jul 21;9(29):eadg0278. doi: 10.1126/sciadv.adg0278 (PMC10361587; doi:10.1126/sciadv.adg0278)
Supplement: Supplementary file 1 — Supplementary Text Figs. S1 and S2 [file sciadv.adg0278_sm.pdf]

Science Advances
AAAS

Supplementary Materials for

# Global-scale random bottom pressure fluctuations from oceanic intrinsic variability

Mengnan Zhao *et al*.

Corresponding author: Rui M. Ponte, rponte@aer.com

*Sci. Adv.* **9**, eadg0278 (2023)
DOI: 10.1126/sciadv.adg0278

**This PDF file includes:**

Supplementary Text
Figs. S1 and S2

# Supplementary materials

**Temporal behavior of $p_b^i$ mode 1** Principal component time series of EOF mode 1 of $p_b^i$ from all ensemble members (Fig. S1) indicate the randomness of intrinsic variability. Any one of them could represent the real ocean $p_b^i$ variability. Large changes over periods of a few months are seen with some variability also present at longer timescales.

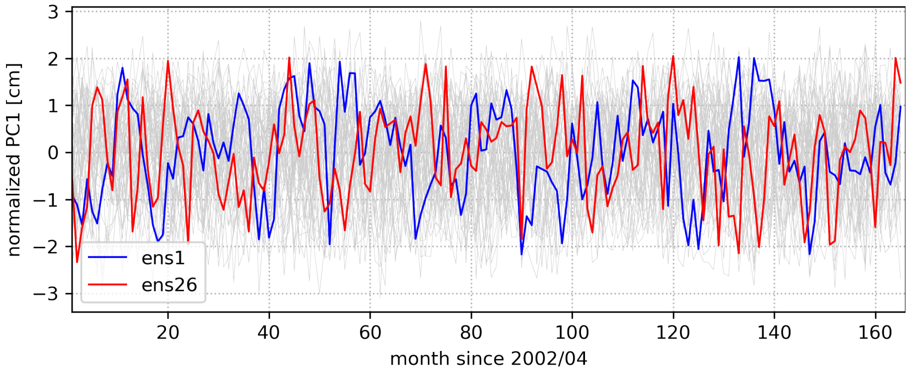


**Fig. S1. Normalized Principal Component time series of mode 1 of $p_b^i$ variability [cm].** Time series corresponding to mode 1 $p_b^i$ of all ensemble members (grey). Colored lines highlight two arbitrarily chosen members.

**Consistency of total $p_b$ variations between GRACE and OCCIPUT** The similarity between standard deviations of total $p_b$ in OCCIPUT and GRACE (Fig. S2) indicates that OCCIPUT simulations are realistic, and gives confidence to the results presented in this study.

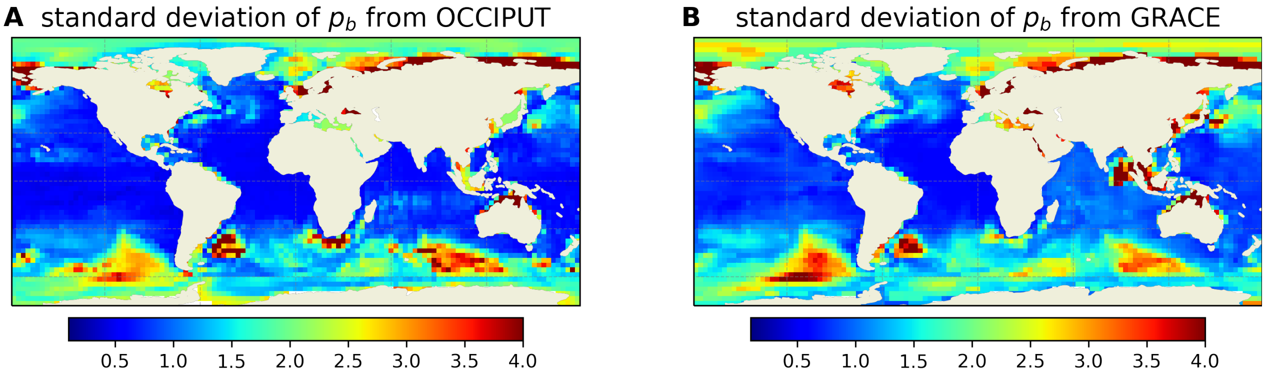


**Fig. S2. Comparison of total $p_b$ variability in OCCIPUT and GRACE.** Standard deviation of total $p_b$ [cm] from one arbitrary OCCIPUT ensemble member (A) and from GRACE (B).
